# Tension Pneumothorax Following Spontaneous Enucleation of a Hydatid Cyst into the Pleural Cavity

**DOI:** 10.1590/0037-8682-0219-2026

**Published:** 2026-09-25

**Authors:** Yener Aydin, Ali Bilal Ulas, Zehra Nur Cavdar

**Affiliations:** 1 Ataturk University, Medical Faculty, Department of Thoracic Surgery, Erzurum, Turkey.

A 10-year-old boy presented to the emergency department with chest pain and dyspnea. A posteroanterior chest radiograph revealed a tension pneumothorax in the right hemithorax, and an emergency tube thoracostomy was performed. Subsequently, the patient had massive air leakage from the chest tube, and follow-up thoracic computed tomography revealed a cavitary lesion in the right upper lobe, and an appearance consistent with a cystic lesion over the diaphragm (Figure 1). A right utility thoracotomy combined with thoracoscopy was performed. The cystic cavity was managed with cystotomy and capitonnage, and the hydatid cyst that had enucleated into the pleural cavity was excised intact. The patient was discharged without complications on the fourth post-operative day.

**FIGURE 1:**
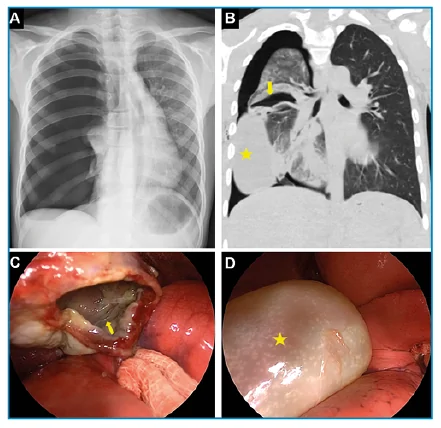
Posteroanterior chest radiograph **(A)**, showing tension pneumothorax in the right hemithorax. The coronal thoracic computed tomography image obtained after tube thoracostomy **(B)**, shows a cystic cavity in the right upper lobe (arrow) and an appearance consistent with an enucleated hydatid cyst over the diaphragm (asterisk). Intra-operative images **(C,D)**, show a cystic cavity in the upper lobe (arrow) and a hydatid cyst over the diaphragm (asterisk).

Hydatid cysts, caused by *Echinococcus granulosus*, are acquired by ingesting contaminated food or water, or by direct contact with infected animals^1^. Pulmonary hydatid cyst rupture can lead to different complications depending on whether the rupture extends into the pleural cavity or into the bronchial system. Pleural rupture may lead to pneumothorax, pleural effusion, or pyopneumothorax, and spillage of cyst contents increases the risk of anaphylaxis^1^ ^,^ ^2^. Bronchial rupture, in contrast, typically presents with a sudden cough, expectoration of cyst membranes, and secondary infection^3^. Very rarely, the pericyst may rupture, and the hydatid cyst may enucleate into the pleura, and the patient may present with a tension pneumothorax.
